# Social Bots’ Role in the COVID-19 Pandemic Discussion on Twitter

**DOI:** 10.3390/ijerph20043284

**Published:** 2023-02-13

**Authors:** Yaming Zhang, Wenjie Song, Jiang Shao, Majed Abbas, Jiaqi Zhang, Yaya H. Koura, Yanyuan Su

**Affiliations:** 1 School of Economics and Management, Yanshan University, Qinhuangdao 066004, China; 2 Internet Plus and Industrial Development Research Center, Yanshan University, Qinhuangdao 066004, China; 3 School of Foreign Languages, Yanshan University, Qinhuangdao 066004, China

**Keywords:** social bots, COVID-19 pandemic, social media analytics, Twitter

## Abstract

Social bots have already infiltrated social media platforms, such as Twitter, Facebook, and so on. Exploring the role of social bots in discussions of the COVID-19 pandemic, as well as comparing the behavioral differences between social bots and humans, is an important foundation for studying public health opinion dissemination. We collected data on Twitter and used Botometer to classify users into social bots and humans. Machine learning methods were used to analyze the characteristics of topic semantics, sentiment attributes, dissemination intentions, and interaction patterns of humans and social bots. The results show that 22% of these accounts were social bots, while 78% were humans, and there are significant differences in the behavioral characteristics between them. Social bots are more concerned with the topics of public health news than humans are with individual health and daily lives. More than 85% of bots’ tweets are liked, and they have a large number of followers and friends, which means they have influence on internet users’ perceptions about disease transmission and public health. In addition, social bots, located mainly in Europe and America countries, create an “authoritative” image by posting a lot of news, which in turn gains more attention and has a significant effect on humans. The findings contribute to understanding the behavioral patterns of new technologies such as social bots and their role in the dissemination of public health information.

## 1. Introduction

The global pandemic of COVID-19 has not only seriously affected the economic situation and people’s lives and health around the world, but has also spawned an “infodemic”. A large amount of unexpected, mixed true and false information has spread rapidly and massively on social media, making panic and anxiety. False information has been widely accepted and even disseminated, forming a misleading and coercive attitude in public’s cognition and ideology. Inaccurate and even dangerous information has been widely spread on social media, crazy conspiracy theories are infecting the Internet, and hatred is spreading virally [1]. This shows that, compared with the “new coronavirus”, the “public opinion virus” is even more terrifying, spreading even faster and wider, and having a stronger impact on public perception [2]. Social bots, as a new intelligent technology, have gradually penetrated the online social media space and even become the agents of these “opinion viruses” [3]. They hide behind social media accounts, integrate codes to simulate humans, and produce and disseminate content related to public health. Some studies have shown that the activities of social bots led to hatred and extremism during the COVID-19 pandemic [4,5].

There is no doubt that social bots have become an important component of complex social networks. They are turning into an “invisible hand” to exert a sophisticated and profound influence on the ecosystem of public health opinion, which is changing from being dominated entirely by people to a new model of “humans + social bots” interactive symbiosis [6]. Social bots are no longer just a medium of communication, but have become the main body of communication [4]. This penetration of social networks is not only a technical means of using algorithms to spread ideology, but also a political behavior that achieves the purpose of manipulating public opinion through propaganda. It has both technical and social attributes. Social bots are becoming more like real people by imitating human user behaviors, participating in public health discussions, and actively interacting with humans based on disseminating a lot of information related to public health.

At present, many studies, especially in Europe and the US, generally believe that the use of social bots has negative effects on public opinion regarding many sensible topics [5,6,7,8]. Some social bots can manipulate online discussions of important issues ranging from elections to public health, threatening the constructive exchange of information. They have mainly focused on the negative behavior of social bots in spreading false information, maliciously manipulating public opinion, and falsifying news. However, social bots are ultimately a technical tool rooted in the underlying social structure, and their positive applications are increasingly being discovered. The convergence of communication shows signs of positive feedback [9]. Understanding the differences between social bots and human personas is of great significance for regulating the application of social bots, governing online public opinion on public health emergencies, and establishing a healthy public opinion ecosystem. Therefore, the purpose of this article is to compare the differences of topics, sentiments, intentions, and interactions between social bots and humans, and to analyze the information dissemination characteristics of social bots and the impact of their interactions with humans. Specifically, we focus on the following research questions:

RQ1a: What is the proportion of tweets posted by social bots and humans in COVID-19 discussions on Twitter?

RQ1b: What topics do social bots and humans focus on in COVID-19 discussions? What is the difference in focus?

RQ2: What are the sentiment attributes of social bots and humans in these discussions?

RQ3: How do the account profiles of social bots and humans differ? How do social bots portray themselves to achieve their communication intentions?

RQ4: How do the communication behaviors of social bots differ from those of humans? What are the communication strategies of social bots?

## 2. Literature Review

### 2.1. Human–Machine Communication Theory

The field of human–machine communication was officially recognized for the first time at the 2016 Annual Meeting of the International Communication Association (ICA). It is of considerable significance to study the interaction and communication between humans and machines and its impact on society [10]. In Andrea L. Guzman’s view, “human–machine communication” refers to the exchange of information between people and machines or technologies designed to realize the function of communicators, and the meaning generation produced by the two in the process of exchange. The core idea is to regard the machine as a communicator and the meaning generation between humans and machines as the focus of human–machine communication research [4,11]. The theory mainly includes three research components.

The first component is the functional orientation: From an instrumental perspective, they discuss how humans use machine equipment and technology, and they regard machines as manipulative tools for human transmission of information. The term bot originated in the 20th century and means “forced labor” or “slave”. This implication reflects a kind of instrumental and object epistemology produced by people. Social bots, according to scholars such as Boshmaf, are intelligent programs that autonomously run social accounts, automatically send information, and link requests in social networks, and are essentially computer algorithms that can realize information dissemination [12]. In this orientation, scholars focus on the technical support role of social bots as communication tools. Theories at this level mainly include the theory of reasoned action (TRA), unified theory of acceptance and use of technology model (UTAUT), technology acceptance model (TAM), and other technology acceptance theories [13].

The second component is relational orientation, which studies the social connection between humans and machines from a media perspective and regards machines as communicators equal to humans. Communication theory in the traditional sense only regards machines as an intermediary and studies communication between people. In 1996, Byron Reeves and others put forward the theory of media equivalence (Media Equation) in their book “Media Equation”, explaining the view that “media is equal to people” [14]. They believe that people will characterize robots as “races” with social attributes and communicate and interact with them based on a series of social cues (such as text, images, voice, video, etc.) generated by the machine, resulting in social connection. As artificial intelligence technology matures, machines will have greater social interaction. The HMC theory subverts the thinking that “technology is only an intermediary”, pointing out that the machine is no longer just an intermediary, but a communicator. Hence, its role in communication should be changed from a channel to a source of information, and the communication behavior should be changed from static to interactive [11].

The third component is the ontology orientation, which studies the blurred boundaries between humans, machines, and communication from the perspective of interactivity. It reflects on the traditional view of “human-centered” and re-examines the relationship between humans and machines in the age of artificial intelligence. This orientation has metaphysical significance. The actor-network theory (ANT) proposed by Latour considers people, machines, communication, etc. as decentralized and equal actors, and they establish connections through mutual translation, thereby forming a real-time change network [15]. This theory emphasizes the non-human attributes of actors and the initiative of non-human subjects, and inspires people to re-examine the ontological relationship between humans and technology in a post-human society that tends to be mediated by technology.

The research on the ontology of the human–machine relationship is becoming more and more flexible at the above three levels, and the initiative of the machine is gradually enhanced. In the context of the rise of natural language processing (NLP) and natural language generation (NLG) technologies, machines no longer only play the role of “media” to assist communication, but gradually become the main body of behavior that dynamically participates in communication [16]. Therefore, it is necessary for us to revisit and even rethink the definition of “communication”. This is first reflected in thinking about the function of the machine as a communicator, drawing on the theories and methods that can be used to study human–machine communication in the traditional communication system, and seeking a breakthrough in the original research boundary. In addition, given that communication is a social activity, communication studies should also understand the significance of intelligent technology as the interaction between communicators and humans at the relational level. Finally, human–machine communication needs to go deep at the metaphysical level, to re-examine the ontological gap between people and technology placed by traditional communication theories [4]. However, as far as current research is concerned, the functional orientation focuses too much on the rationality of tools, which is not conducive to exploring the intelligence of social bots. Therefore, we adopt a relational approach, regard machines as communicators equivalent to humans, and study the communication behavior process and social relationship interaction established between humans and social bots.

### 2.2. Social Bots

With the development of artificial intelligence and big data technology, social bots are playing an important role, they have become a widely accepted force in social media platforms [17]. Boshmaf et al. defined the social bot as an intelligent program that can autonomously run social accounts in a social network and have the ability to automatically send information and link requests, emphasizing its automated behavior of disseminating information [18]. Woolley and Howard further pointed out that social bots simulate real users and produce informational content by integrating codes with the purpose of manipulating public opinion and disrupting organized communication [19]. Although their views are not completely unified, scholars generally agree that the essence of social bots is a kind of computer intelligent algorithm in social networks [20], and their actions are hidden behind the will of human operators [18]. Social bots are often associated with fake news and computational propaganda. The use of social bots to spread false information is the basic way to carry out computational propaganda. They can automatically generate contents and interact with humans on social networks [21], trying to imitate and even change human behavior [22]. Scholars have carried out research on the dissemination contents, behavior mode, dissemination scale, and social impact of social bots.

In terms of dissemination content, social bots can be divided into four types: government agency control, politician-party control, business company participation, and social group cooperation. In fact, 76% of countries in the world have confirmed that their government agencies/politician parties use social bots to guide public opinion and participate in political competition. Thus, social bots are mostly controlled by politicians with sufficient resources and power to speak [23]. It has become an important factor in interfering in online elections. Social bots spread large amounts of information to achieve their purpose: supporting political parties, attacking opponents, suppressing dissidents, and creating differences [22]. Studies have shown that social bots account for 9–15% of active Twitter accounts [24]. In some specific topics, the proportion of published content can even reach more than 50% [25].

In terms of behavior patterns, social bots have behavioral models, such as imitating human behavior, promoting information diffusion, and interacting with humans. Human mimicry refers to social bots attempting to mimic all online activities of real users to make themselves appear human [26], including behaviors that expand their own influence [27], infiltrating utterances behavior in discussion [28] and behavior in building social relationships [5]. Although some researchers have devoted themselves to the identification and detection of social bots, a unified standard has not yet been formed in the academic community [29,30]. Promoting information diffusion behavior refers to the key role that social bots play in information production and diffusion [31]. Most scholars pay attention to the negative effects of malicious misuses of social bots, including social bots’ behaviors of manipulating public opinion, baiting, and information sharing strategies [32], and actively generating and spreading politically inclined information [33]. Scholars have found that the behavior of social bots to promote information diffusion not only acts on negative information [34], but also acts on positive information [35]. Human–bot interaction behavior refers to the interaction between social bots and humans, which in turn affects their own and human behavior. Whether through direct dialogue [36] or indirect interaction [6], these interactions with humans create a trustworthy “persona” for them. At present, social bots have successfully penetrated social networks, and their penetration success rate on Facebook has reached as high as 80% [37].

In terms of the scale of dissemination, the scale of social bots’ application promotion can be divided into three levels [19]. In the first level, the number of teams is small, limited to the primary stage of domestic or political election operations. This level is common in Argentina, Germany, Italy, the Netherlands, Greece, Spain, Sweden, and South Africa. In the second level, there are a certain number of full-time personnel who use a variety of strategic tools to coordinate with the intermediate stage of long-term control of public opinion and even influence overseas. This scale is found in Austria, Brazil, Czech Republic, Indonesia, Kenya, Malaysia, Mexico, South Korea, Thailand, etc. The third scale is the advanced stage of domestic and overseas dissemination (this is common in the United States, the United Kingdom, Russia, Australia, Egypt, India, Israel, Ukraine, Saudi Arabia, the Philippines, and other countries), with many team members and plans to invest in R&D operations.

In terms of social impact, the personalization characteristics of social bots are becoming more and more obvious, their impact on the ecology of public opinion is also becoming more complex and profound. As a “new specie” active in the network, it influences the public’s cognitive behavior by interacting with humans, like an “invisible hand” influencing online political public opinion and the real world. During the Brexit referendum, some networks composed of a large number of social bots frequently posted relevant information on Twitter and disappeared shortly after the referendum [38]. During the 2016 US presidential election, social bots were more active in the center of social networking [39]. In addition to political events, discussions of public topics, such as vaccination [40], social unrest events [41], and global warming [42], have also been found a robot navy with highly coordinated behavior. The social influence of social bots continues to expand with the continuous maturity of algorithm technology and the needs of operators.

### 2.3. User Persona

With the rise of social media platforms and the continuous surge in the number of users, massive user personal information and behavioral data are left on the platforms [43]. This provides a continuous impetus for social media user profiling research [44]. As a virtual representation of the user’s real data [45], user personas have become a current research craze. Some studies constructed a user persona model based on user interests in order to provide more accurate personalized recommendations to users [46]. Using Facebook user keywords as a data source, they established a similarity function to quantify the similarity between users and find the affected similarities in social media platform dating [47]. Some studies used clustering methods to analyze users’ behavior logs on the platform and constructed a user behavior persona model [48]. Some other studies explored the construction and application of user persona in personalized recommendation systems [49]. For example, they constructed user persona based on the time users spent using a social media platform and browsing behaviors and used them to recommend articles of interest to users [50]. Alternatively, they build user persona models by mining voting data from social media users and optimizing dynamic personas based on user feedback [51].

Most of the objects and data sources of user persona research come from real users. However, with highly anthropomorphic characteristics, social bots are active in social networks, and their behaviors have both the social attributes of traditional users and the technical attributes of intelligent algorithms. While it is not an actual “netizen” in the traditional sense, it is a social media user. Moreover, few studies constructed user persona for social bots based on a comparative analysis between social bots and human users. Therefore, we use the theory of user persona and propose the concept of social bots’ persona. The aim of this research is to study the characteristics of social bots, an active entity used in the dissemination of social media information, as well as its role in the discussion of public health topics. It benefits in accurately understanding the behavioral characteristics of social bots in public opinion dissemination during public health emergencies, allowing for better supervision and governance of social bots.

## 3. Data and Methodology

**Figure 1:**
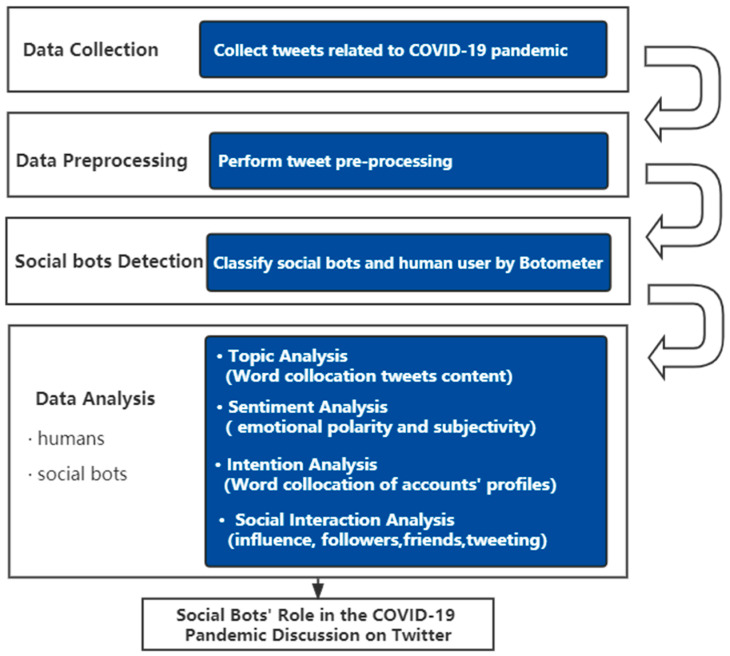
Overview of research structure.

A computational approach combining text analysis, sentiment analysis, and social network analysis was utilized to answer the research questions (The overview of the research structure is shown in Figure 1). Our method assumes that both social bots and humans share their opinions, attitudes, and feelings by disseminating information on social media platforms. These emotions and ideas are represented in short sentences that include keywords and words that represent their hidden beliefs and attitudes.

During the preprocessing step, we remove extraneous words (non-index words) in order to find the major themes and terms in the text data. The non-index word-iso list comprising unneeded English words is utilized to filter the unnecessary terms in this method. Furthermore, before text analysis, the emoticons and links were removed from the data to clarify the text discovery gained from the tweets. In our study, terms and words were employed to represent the underlying drivers of the features and behavior of social bots and humans during the COVID-19 pandemic. Firstly, we identify keywords in a collected sample of tweets. To identify the topics of discussion, we utilize text summarizing algorithms. Secondly, we measure the emotional polarity and emotional subjectivity of social bots and humans. Next, we analyze social bots’ intentions and motivations for disseminating information based on their user profiles. Finally, by using the n-gram text tokenization technique [52], which is commonly utilized in text mining and natural language processing (NLP) [53], we analyze account profiles of social bots and humans, and obtain commonly used keywords and expressions to determine their propagation intentions and interaction processes. This section provides a detailed explanation of our methodology.

### 3.1. Data Collection

**Figure 2:**
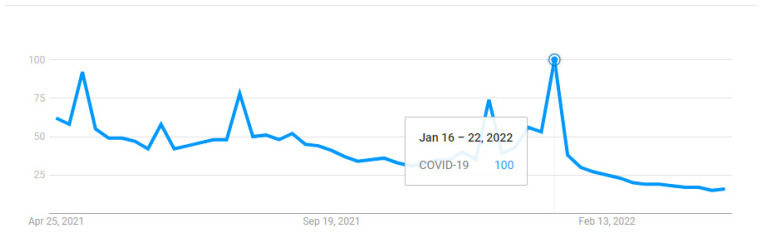
Google trends of research interest related to COVID-19 pandemic.

Among social media platforms, Twitter is a popular public communication network that provides instant access to a vast amount of bottom-up created data covering various topics of interest. Researchers are considering social media particularly Twitter for various studies due to the rich spatial, temporal, and social attributes of these data. Therefore, Twitter was chosen as the data collection source. Besides tweets, information about time, location, and users is also attached, which is beneficial for a variety of research reasons. Social media users often anchor their tweets to topics with specific hashtags, thereby triggering users who follow the same topics to engage in online communication and public discussion on beaconed topics. Therefore, we created a search application using the Python package Tweepy to collect relevant tweets. Twitter provides an application programming interface (API) for browsing and streaming tweets made by users. The API allows searching for tweets produced at a given time by setting the start and end search dates, ensuring that the tweets are searched within one week of the search time. Users can look for tweets based on their appearance or keywords. The search criteria may be restricted to just capturing tweets with specific characteristics (such as tweet language). Searching also allows retrieving specific fields of a tweet instead of the entire tweet. Due to the long time period of the COVID-19 pandemic, we identified significant time points in recent years by searching Google Trends for the keyword COVID-19 (as shown in Figure 2). We found that social media users continued to follow topics related to the COVID-19 pandemic, and that the focus largely covered all countries around the world. Therefore, we used #COVID-19 as a key topic and used the Twitter Standard Search API to identify tweets about the COVID-19 pandemic outbreak in English.

We set up the data collection cycle with two main principles. Firstly, we selected data from the peak period of social media user discussion, which is a large and representative period, and where social bots produce and disseminate a large amount of information. The public interest in the COVID-19 pandemic was once again at its peak. Secondly, due to the existence of an anti-bot mechanism on the Twitter platform that monitors and blocks bot accounts in real time [54], we kept the data collection time to a short period (7 days) in order to reduce the research bias caused by Twitter blocking. When these two conditions were met, we followed [55] to use convenience sampling and set the most recent discussion peak period as the data collection cycle, with 16 January 2022 as the start date and 22 January 2022 as the end date. We collected a total of 56,897 tweets and pre-processed 54,919 tweets as the study sample, which include user-name, country, date, content, reply_count, retweet_count, like_count, quote_count, friends_count, follower_count, etc. Then, we combined the data crawl fields to modify the code and crawl the relevant data. We ran the program several times over the sample period to retrieve all relevant tweets.

### 3.2. Data Preprocessing

A tweet is a type of text data that includes a lot of acronyms, numbers, slang terms, and hyperlinks. Using the RE library, we created a Python program to delete numbers, hash tags, and hyper-links. Then, we eliminated tweets with fewer than 80 characters and repeated tweets. To cope with slang terms and often used abbreviations, we developed another Python program that substitutes an abbreviation with its complete meaning by referring to a manually produced text dictionary. Entries are easily accessible, and new terms and abbreviations with their related meanings may be added. Throughout the study period, acronyms and terms were replaced to increase the accuracy of keyword selection and factor discovery. At last, the sample was saved as a file to be processed on other platforms.

In the preprocessing step before analysis, we utilize the regular expression library in Python to remove any non-alphabetic characters, including tags, URLs, and punctuation, and replace spaces with single spaces. After deleting the non-alphabetic letters, we split the compound word spell checker library in Python to fix any misspellings. In addition, emoticons and links were also deleted from the data prior to text analysis to clarify the textual conclusions generated from tweets. Following data cleaning, the terms in each tweet are tokenized, and a composite set is formed. Next, we may restrict the composite set in each tweet to certain elements of speech, such as verbs, adjectives, nouns, or adverbs. This helps to improve the efficiency, efficacy, and adaptability of word collocation. Finally, we eliminate stop words using the NLTK Python package, which is essential for locating unique words in text.

### 3.3. Social Bots Detection

By combing through existing studies, we used Botometer (a project developed by OSoMe and IUNI at Indiana State University, USA) to perform social bot identity checking [5,56]. Botometer is a trained machine learning algorithm that extracts over a thousand features by tagging tens of thousands of example accounts and can be used to characterize information such as profiles, friends, social network structure, temporal activity patterns, language, and sentiment in accounts [26]. When detecting an account, Botometer uses Twitter API to obtain a public profile of the account and transmits it to Botometer API, which compares an account to tens of thousands of already-tagged accounts and finally scores the account. The closer the score is to 1, the more likely the account is a bot account. The closer the score is to 0, the more likely the account is a human account. Some studies have identified accounts as belonging to a bot when the score is greater than or equal to 0.3 [8], and more studies have identified accounts as highly suspected bots when the score is greater than or equal to 0.5. We followed most of the previous studies [5,33,56] and labeled accounts with bot scores greater than 0.5 as social bots and accounts with bot scores below 0.5 as humans. Botometer may not give bot scores for accounts due to various reasons, such as authorization issues, account suspension, and lack of tweets for analysis. The identity of these accounts is recorded as “Unknown”. Unknown users contributed 2.39% of all tweets, and these accounts were excluded from the data analysis. We collected 54,921 tweets and randomly selected 1000 from the data, finding that 97% of them were related to the COVID-19 pandemic, indicating that our data are suitable for analysis.

### 3.4. Keyword Selection

We use R to select keywords because it has a powerful library for summarizing tweet content. In particular, the udpipe library contains pretrained models for word assembly, keyword selection, and text annotation. The document term matrix (DTM), which contains all the words used in the tweet, was formed after we annotated the text using the udpipe R library to obtain the lemmas and part of speech tags. Stop words and low frequency words can be excluded using the document term matrix function in the TM library in R. We decide to remove terms that are used in less than 1% of all tweets. In DTM, each row corresponds to a tweet, and each column corresponds to a term. When the tweet represented by the row I contains the word w, which is represented by the column j, then wij = 1; otherwise wij = 0. Then, we remove all other words to keep only nouns.

### 3.5. Word Collocation

The main topics of discussion in the sample are represented by terms that are highly collocated and frequent. Word collocation refers to the recurrence of words together. We selected a pre-trained machine learning model that extracted keywords and word collations using the udpipe library in R Studio. The 50 most frequent words in collocation are displayed in a single undirected relationship between nodes representing collocating terms. We can identify the main themes for social bots and humans by reading these terms and examining their relationships.

### 3.6. Sentiment Measure

We divide tweets into two categories based on social bots and humans. Then, the Textblob Python library, which is based on the lexical sentiment analysis methodology, is used to calculate the sentiments for each category. Based on the predetermined sentiment value of the terms that make up the document, Textblob determines the sentiment polarity and subjectivity of the document. We measured the average sentiment polarity and subjectivity of tweets posted by social bots and humans on COVID-19 pandemic topics, respectively, and plotted the distribution of these sentiments. As the characteristics of that distribution reflect differences in sentiment, we pay more attention to the sentiment distribution than the average.

## 4. Results and Findings

### 4.1. Basic Statistics Analysis

**Figure 3:**
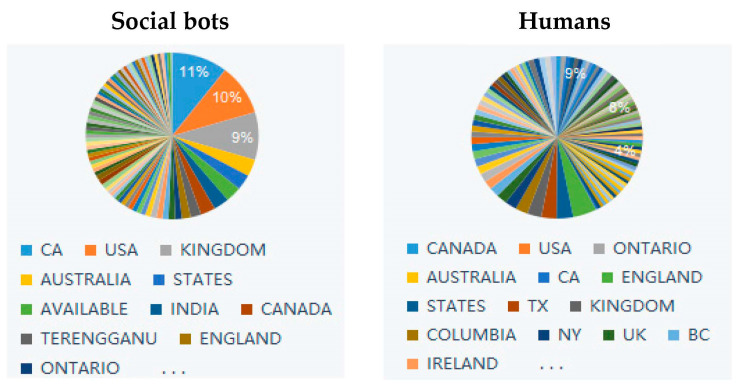
Tweet’s location of social bots and humans.

We conducted the analysis on a sample of 54,919 tweets, 14,499 tweets (26.4%) originated from social bots, 38,774 tweets (71.21%) from humans, and there were 1648 (2.39%) tweets for which the bot score was not available, so we set them as unknown users (not included in the analysis), resulting in 53,608 tweets being included in the analysis. The average text length of these tweets was 110, slightly longer for human users than for social bots. By analyzing the location of accounts, we found that humans and social bots accounts are located in similar regions, mainly in Canada, United States, United Kingdom, Australia and India (as shown in Table 1). However, the geographical distribution of human accounts is more dispersed, while the regional distribution of social bots is more concentrated, with more than 30% of social bots’ accounts located in Europe and the United States (as shown in Figure 3).

**Figure 4:**
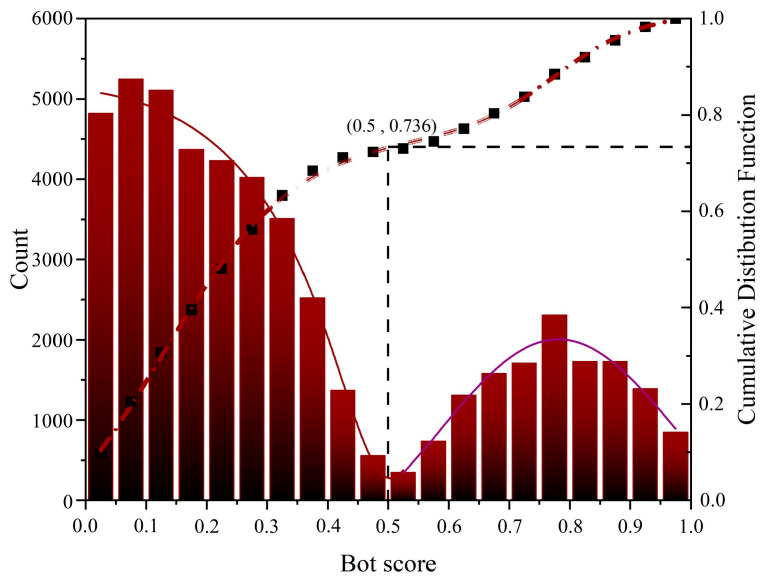
Frequency distribution and cumulative distribution of Twitter account bot score.

We visualize the overall distribution of all user bot score detections through frequency distribution histograms and cumulative distribution functions (as shown in Figure 4). Among them, the average bot score of all accounts is 0.35, with 0.03 mode, and 0.26 median. The average level of all the accounts is close to the upper tertile, and the distribution and aggregation boundaries between humans and social bots are relatively distinguishable. Taking bot score = 0.5 as the dividing line, non-social bot accounts (score below 0.5) accounted for 73.6%, and social bots accounted for 26.4%. To a certain extent, it also shows that using 0.5 as the criterion for distinguishing between human and social bot accounts is suitable.

### 4.2. Topic Analysis

**Figure 5:**
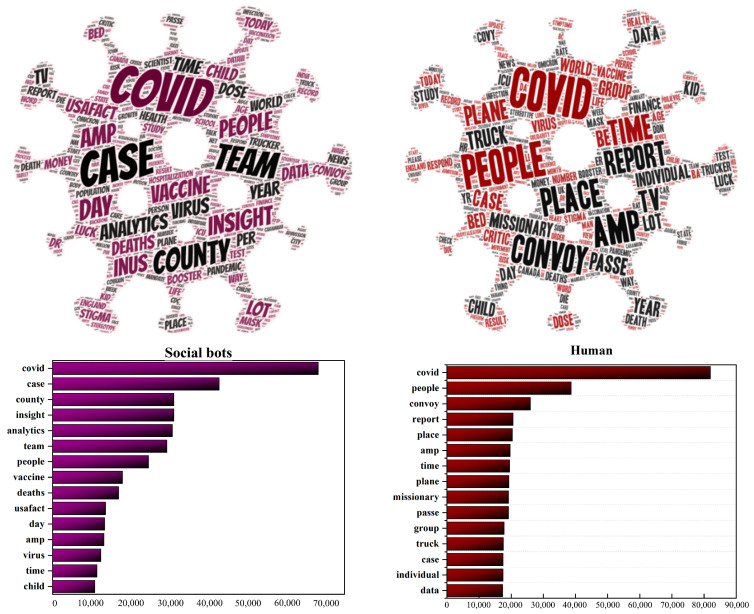
Word cloud and word frequency distribution of tweets posted by social bots and humans.

Using natural language processing, we analyze high-frequency words, topics, and sentiment of tweets posted by social bots. We use R language to process the raw data. By analyzing tweets content, we find that social bots and humans focus on COVID-19 pandemic and they both seem quite passionate about this topic. However, the focus of the two is also different. Specifically, humans are more concerned about topics that are closely related to human beings, such as people’s lives, inter-city mobility, and drug treatment. Words, such as “people” and “convoy”, have become the focus of humans. Social bots are more concerned with news topics related to the real-time evolution of the epidemic, such as confirmed cases, mortality, data analysis, and vaccine research. Words, such as “case”, “country”, and “analytics”, are the focus of social bots. Figure 5 shows the word cloud and the top 15 high-frequency words of the contents published by humans and social bots. Topics for humans are scattered and evenly distributed, while topics for social bots are ladder-like, which shows that the topics that humans care about are broad and scattered, while the topics that social bots care about are relatively few and clustered.

**Figure 6:**
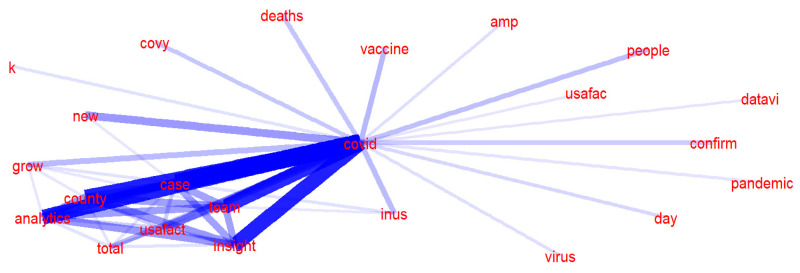
Word collocation of tweets posted by social bots.

**Figure 7:**
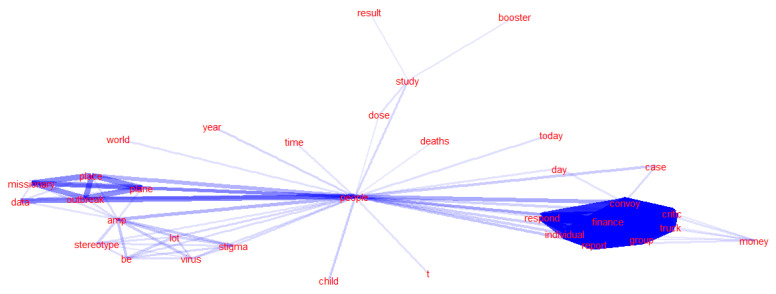
Word collocation of tweets posted by humans.

Figure 6 and Figure 7 present the word collocation of the top 50 frequent words in the sample of tweets of social bots and humans. Comparing the two figures, the text content published by social bots is centered on “COVID-19”, and they widely pay attention to various topics related to the news about the evolution of the pandemic. These topics mainly revolve around “disease” and “virus” and are closely related to each other. As shown in Figure 3, words, such as “case”, “country”, “analytics”, “team”, and “USA Facts”, frequently appear in their tweets, which are the focus of discussion among social bots. They are concerned about the real-time data of confirmed cases, mortality, and other epidemic situations in various countries, as well as the overall trend of the development and changes of the pandemic. They pay attention to the real-time data of USA Facts and the COVID-19 vaccine research progress. The topics they care about are closely related to the pandemic itself, and most of these topics are objective and widely disseminated through the news media. On the other hand, the text content published by humans is centered on “people”, and they pay more attention to various secondary topics. These topics are quite different, covering various fields, such as the economy and finance, social order, medical and health, but they are all closely related to people’s lives. As shown in Figure 7, there are three subtopics that have gained more attention. Humans are mostly concerned about the living conditions of individuals under the influence of the epidemic, inter-city population mobility policies, immigration status, and social and economic development.

### 4.3. Sentiment Analysis

We use Python to run the TextBlob library to perform sentiment analysis on the content of tweets posted by social bots versus humans. It categorizes text into a range from negative to positive, with neutral in the middle. Sentiment is measured primarily through two indicators: polarity and subjectivity. In TextBlob, polarity indicates how negative or positive a sentiment is, expressed as a floating-point number, ranging from −1.0 (negative sentiment) to 1.0 (positive sentiment), with 0 representing neutral sentiment. Subjectivity indicates whether an emotion is based on a trusted or verifiable source or if it is simply an opinion, an emotion, or a judgment. It is represented as a floating value between 0.0 and 1.0, with 1 representing a more subjective (opinion-based) sentiment.

**Figure 8:**
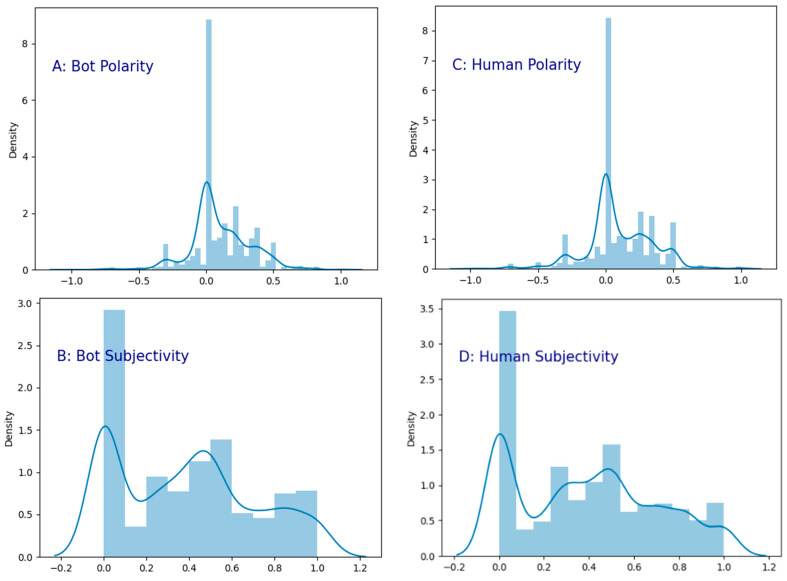
Sentiment analysis of content posted by social bots and humans on Twitter.

As shown in Figure 8, the distributions of emotional polarity and subjectivity between social bots and humans are remarkably similar. From the perspective of emotional polarity, the user group with neutral emotions (a score of almost 0) accounts for the largest proportion. Most of the other users have negative emotions. The floating value is basically between 0.0 and 0.5, and the number of positive emotions is relatively small. The floating value is basically between −0.5 and 0, and there are almost no users with extreme emotions. Comparing the sentiment research in the COVID-19 pandemic discussions on Twitter when the crisis first broke out [57], in the early stage of the epidemic, a large number of social media users panicked, worried, and expressed other negative sentiment, and social bots spread a lot of negative information, prompting people to infect others with negative sentiment. However, with the passage of time and public awareness of the COVID-19 outbreak, the overall negative sentiment of social media users, including social bots and humans, has gradually decreased. More and more users are maintaining a neutral attitude, with some even expressing a positive emotion. From the perspective of subjectivity, the user group with the highest proportion of objective sentiment (near-zero scores) has the lowest proportion, and the scores of other users range between 0.0 and 1.0. The distribution of subjectivity between social bots and humans is relatively balanced.

### 4.4. Intention Analysis

Whether humans or social bots, users will use social accounts to post and disseminate information on social media platforms. In general, a social account profile depicts a personal image of the user in terms of basic information, areas of interest, hobbies, and interests. Social bots will intentionally falsify their image by enhancing their profile. For example, they try to present themselves as experts in a certain field or disguise themselves as active humans to gain the attention and trust of more followers, in order to guide public opinion or even manipulate it. Therefore, we crawled the profile data of these humans and social bot accounts and conducted a comparative analysis through semantic mining to understand their different focuses in terms of personal image design, main areas of interest, and hobbies, and further analyzed the disguise strategies and communication intentions of social bots.

**Figure 9:**
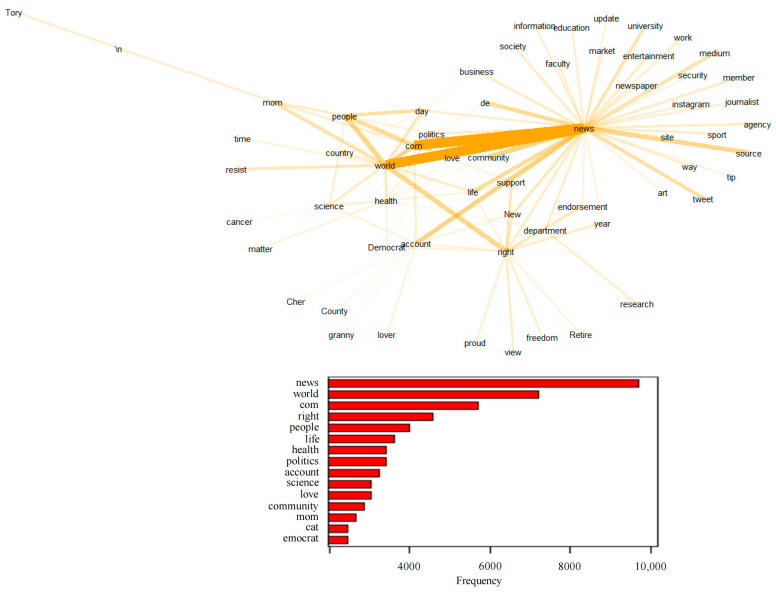
Word collocation of social bot accounts’ profile.

As seen in Figure 9, the account profiles of the social bots prominently feature the words “news”, “world”, “politics”, “right”, and some links. Although the topics they concentrate on seem to be scattered, it all revolves around politics and news from around the world. Most of these social bots use the logos of specific organizations as their avatars in an attempt to create an atmosphere of “authority”. They try to portray themselves as official media organizations, focusing on international issues, posting a lot of news from multiple perspectives, and spreading them around on all platforms. This means that the social bots are posing as objective and political news publishers, with the explicit goal of influencing public opinion and manipulating politics.

**Figure 10:**
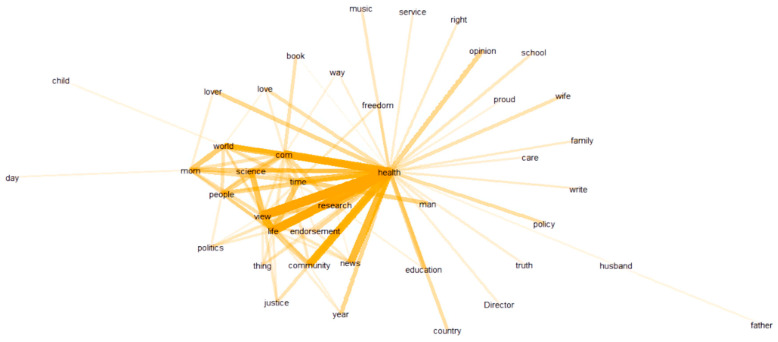

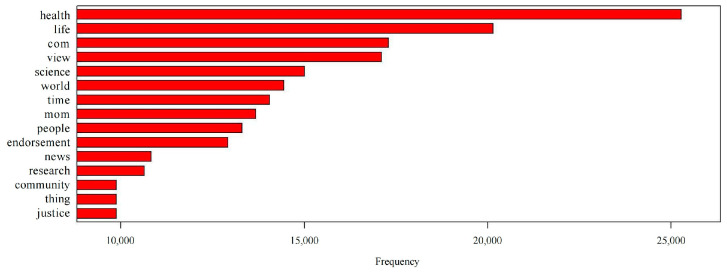
Word collocation of human accounts’ profile.

As shown in Figure 10, humans are more concerned with “health” and “life” in their user profiles than social bots are with “news”. In the context of the COVID-19 pandemic, they are concerned about health, their own health status, the spread of the epidemic around the world, and the progress of research into drug treatments. At the same time, education, family, and social issues are also areas of daily interest to humans. This shows that the communication intentions of social bots are more political, while the image of humans is more lifelike. There are significant differences in their marketing management. It should be noted that even when people are aware that social bots are disguising themselves as the accounts of certain official bodies, it is still very difficult to identify which accounts are social bots and which are the real official authority accounts.

### 4.5. Social Interaction Analysis

(1) Influence

In order to study the social behavior of social bots and humans and their influence we analyze the retweet, like, and reply of tweets published by these two types of accounts. As shown in Table 2, a total of 10,098 social bot accounts (22%) and 35,492 human accounts (78%) were obtained. Among them, more than 90% of social bot and human accounts have been retweeted. However, tweets published by human accounts have been retweeted 1220.74 times on average compared to 1581.76 for tweets published by social bots. This reveals that the information posted by social bot accounts is more likely to be retweeted in large numbers. In terms of like counts, the proportion of social bots is 85.03%, compared to 8.57% of humans. This means that the content about public health published by social bot accounts is more able to attract the attention of social network users and has won the recognition and attention of a lot of users, which also means that they have influenced these users to a certain extent. However, from the perspective of the average number of likes, humans have a higher average than social bots, which shows that human opinion leaders in social networks get more attention. Regarding replies, the proportion of social bots getting replies is lower than that of humans, which shows that social bots tend to interact with likes and forwards. In terms of the coverage of interactive behaviors, the proportion of social bots covering all three behaviors is higher than that of humans. This shows that social bots are more active.

(2) Followers

**Figure 11:**
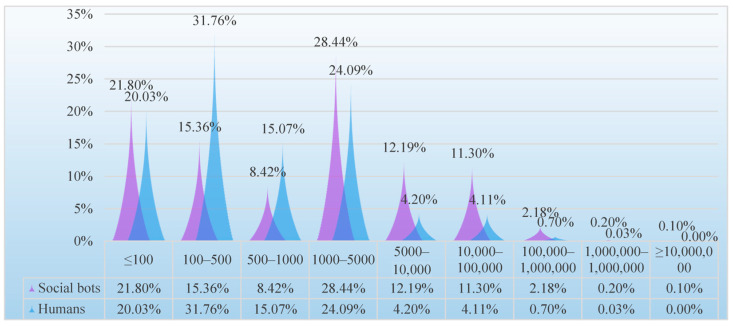
Distribution of the number of followers of social bots and humans.

The average number of humans’ followers is 3788.02, and the average number of social bots’ followers is 629,052.08 (the former is 166 times that of the latter). As shown in Figure 11, the number social bots’ followers is the highest in the range of 1000–5000, while the number of humans’ followers is the highest in the range of 100–500, and more than 50% of humans have fewer than 500 followers. Except that the number of humans’ followers in the 100–1000 interval is higher than that of the social bots, in other intervals, social bots have more followers. Especially in the interval above 5000, the number of social bots’ followers is higher than that of the humans. Moreover, one social bot account is a top account with more than ten million followers, while the number of humans in this interval is 0. The number of social bots’ followers is much higher than that of humans. This shows that although social bots do not have a real interpersonal network like humans, they can imitate human behavior to gain more followers’ attention and even become opinion leaders to lead the direction of public opinion.

(3) Friends

**Figure 12:**
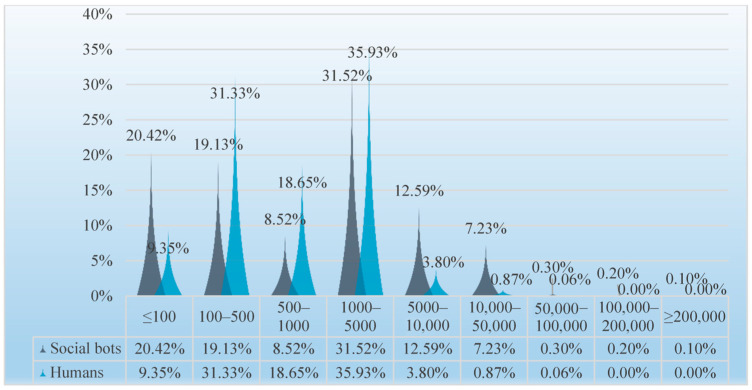
Distribution of the number of friends of social bots and humans.

The average number of humans’ friends is 1537.49, and the average number of social bots’ friends is 21,723.1; the former is 14 times that of the latter. Although it is not as disparate as the difference in the number of followers, it also shows that the number of friends of social bots is much higher than that of humans. As shown in Figure 12, social bots with less than 100 friends are significantly greater in number than humans. In the range of 100–5000, the proportion of humans’ friends is higher than that of social bots’ friends, while in the range above 5000, the proportion of social bots’ friends is higher. Both accounted for the largest proportion in the range of 1000–5000. In summary, the distribution of the number of humans friends is relatively concentrated, mainly in the range of 100–5000. The number of friends in social bots is relatively scattered. Nearly 40% of social bots have less than 500 friends, but they also have accounts with more than 200,000 friends, while the maximum number of friends in humans does not even exceed 100,000. On social media platforms, the number of friends can reflect the influence of users to a certain extent. According to previous research, politicians will buy a lot of bots to greatly increase their number of fans, thereby enhancing their influence in the field of public opinion [58]. Therefore, in COVID-19 pandemic issues, social bots also gain attention by adding a lot of friends.

(4) Tweeting

**Figure 13:**
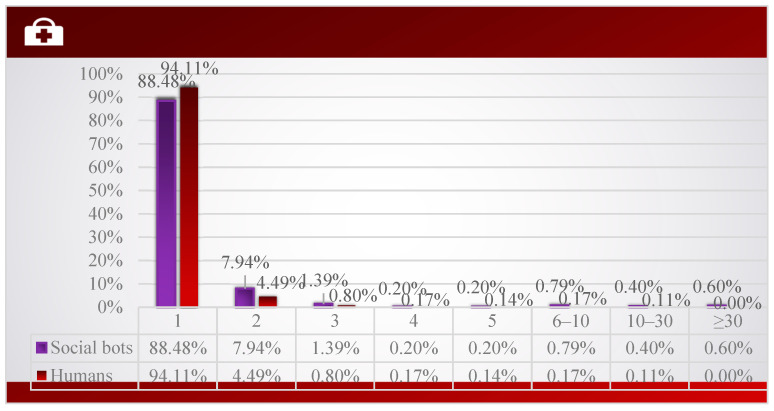
Distribution of tweets number of social bots and humans.

As shown in Figure 13, whether it is social bot or human, most accounts only post one tweet. However, comparing the posting volume of the two, it can be found that the proportion of social bots in the accounts that posted more than two tweets is higher than that of humans, and 0.6% of the social bot accounts posted more than 30 tweets, while humans did not. Therefore, social bots are more likely to post multiple tweets to participate in the discussion of COVID-19 issues. After further reading the tweet content posted by these accounts, we found that social bots often post very similar content related to public health, such as the growth rate of cases and epidemic data in different regions, including similar content posted by the same account and similar content posted by different accounts. This explains that social bots achieve the effect of expanding their influence by copying similar content.

## 5. Discussion

**Figure 14:**
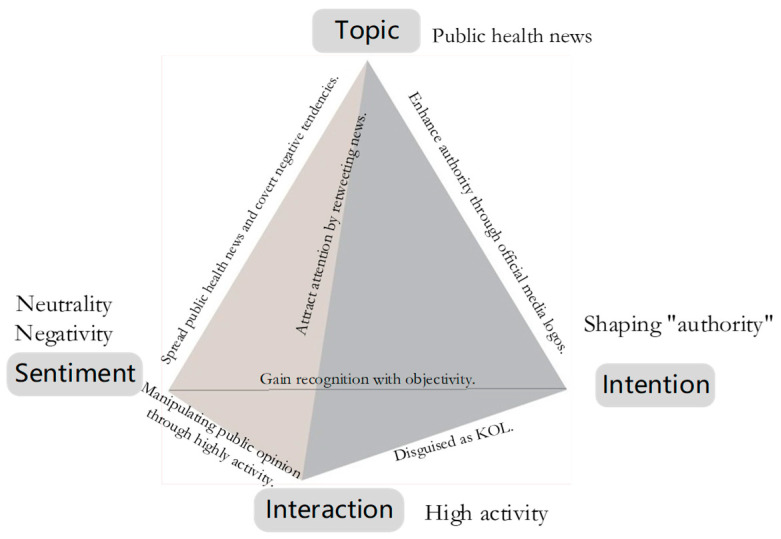
User persona of social bots in COVID-19 pandemic discussion on Twitter.

Studies have shown that social bots appear in discussions on public health issues and hot social events, such as discussions on measles vaccination, discussions on US elections, Brexit, and Sino-US trade negotiations [59,60]. According to the analysis results, social bots are also active in the discussion of topics related to COVID-19 pandemic. Moreover, 22% of these social media accounts participating in the discussion were social bots and 78% were humans. Social bots are not only content dissemination channels and intermediaries, but also participate in the process of discussions on public health topics on Twitter from multiple perspectives. We built user persona for social bots in public health issues (COVID-19 pandemic) after comparing and analyzing the differences between social bots and humans in terms of topics, sentiment, intention, and interaction (Figure 14).

Regarding the topics of tweets, previous study pointed out that humans express more opinions related to themselves, while tweets issued by social bots contain more information [25]. In our study, the tweet topics of social bots focused on news related to the COVID-19 pandemic, such as confirmed cases, death rates, data analysis, vaccine research, etc. The topics of human tweets considers “people” as the center, and they pay more attention to health and life, such as the medical treatment of individuals, the population flow policy between cities, the status of immigration, and the social economy. Additionally, we discovered that humans had an even distribution of keywords, whereas social bots had a stepped distribution. It indicated that humans care about a wide range of topics, while social bots are concentrated on public health discussions and related contents. This feature enabled them to disseminate large amount of news related to public health in a concentrated manner during the epidemic crisis.

In terms of sentiment analysis, previous studies have demonstrated that in the early stages of the crisis, social media users on Twitter primarily showed negative sentiment, such as panic and fear, when discussing topics related to a major epidemic. Meanwhile, social bots spread a large amount of disinformation and prompted negative sentiment [57]. We conducted our research during the second year of the COVID-19 pandemic, and we realized that among social bots and humans, users with neutral sentiment accounted for the largest proportion, the other users mostly had negative sentiment, and the number of those with positive sentiment was the least. This implies that, as time passed and with the public awareness of the COVID-19 pandemic, the overall negative sentiment of social media users, including social bots and humans, gradually decreased, and an increasing number of users tended to maintain a neutral attitude, and a small number of users even held a positive sentiment. This also confirms the neutral attribute of social bots, which have no negative connotations as they represent an emerging technology in social media.

From the perspective of intention, studies have revealed that social bots can exert influence through strategic interactions with influential humans [60]. Many social bot accounts pretend to be news outlets to stay active and attract a lot of attention [61]. According to our findings, social bots attempt to brand themselves as “influential people” in addition to interacting with humans. In their account profiles, they tried to portray themselves as objective and political news publishers with obvious intention of public opinion manipulation. In contrast, humans presented as ordinary. They pay attention to individual health, the research progress of drug treatments, the global spread of the epidemic, and daily issues, such as education, the economy, and society. To some extent, once social bots pose as authoritative organizations and disseminate disinformation, they will manipulate public perception and have a negative impact on the public health environment.

As observed from the interaction analysis, social bots can penetrate social networks to interact with other users. As Boshmaf pointed out, the success rate of social bots’ penetration on Facebook is as high as 80% [18]. We also discovered that, compared with humans, tweets from social bots are more likely to be retweeted in large numbers, as well as receiving a significant amount of likes and attention. Although their capacity to reply is limited, the proportion of retweeted, likes, and replies is higher than that of humans, indicating that they are more activate and efficient in communication. They frequently tweet similar content, including tweets from the same and different accounts, and they increase their influence by copying similar content. Social bots also increase their followers by following other users to gain more attention. It is apparent that social bots are highly active, and if they are used to disseminate health information, they may contribute to the dissemination of public health information during crises.

## 6. Conclusions

As a new technology, social bots have infiltrated the discussion of public health on Twitter. They are highly active and can easily influence public perceptions. If they are used to disseminate health information, they may contribute to public health and the environment during crises. Therefore, it is of great significance to study the phenomenon of social bots’ communication on social media. We collected and analyzed tweets related to the COVID-19 pandemic to compare the differences between social bots and humans in terms of topics, sentiments, intentions, and interactions.

Analyzing 54,919 tweets, we found that social bots were actively engaged in public health discussions on Twitter and contributed up to 22% of accounts. They are mainly located in Canada, the United States, the United Kingdom, Australia, India, and other European and American countries.

Social bots focus on “news” related to public health such as cases, death, data analysis, and vaccine. While humans are more concerned with “people” and engaged in discussions about financial, social, and medical topics related to individual health. Specifically, social bots focus on objective information dissemination. They utilize news as an entrance point to participate in discussions, primarily on topics such as “diseases” and “viruses”, whereas, humans express subjective opinions. They pay attention to various secondary topics derived from “disease” and “virus”, and the topics have a wide range of radiation and great differences.

Social bots and humans have similar distributions of emotional polarity and subjectivity. Users with neutral sentiment accounted for the largest proportion, the rest had negative sentiment, and few users had positive sentiment. The distribution between social bots and humans is relatively balanced. This indicates that social media users’ negative sentiment has gradually decreased with an improved understanding of public health and social network information.

Social bots’ profiles are more “political”, whereas humans’ profiles are more “daily”. Social bots tend to display an “authoritarian image” in order to influence or even manipulate public recognition and opinion. They intentionally create a rich profile to present themselves as an expert in the field of public health and the environment. They also use the logos of some institutions as their avatars, portraying themselves as objective and political news publishers to gain more trust and attention to increase their influence.

Social bots interact with humans through actions such as liking, retweeting, and replying. Their tweets were retweeted and liked at a much higher rate than those of humans. The average number of followers of social bots is 166 times higher than that of humans. Similarly, the average number of friends of social bots is 14 times higher than that of humans. This suggested that social bots are more interactive. Their main strategies are: imitating human behavior via liking and retweeting to attract more attention; gaining friends and followers by following other users; attempting to become an opinion leader by posting a lot of tweets; expanding their public opinion territory by copying similar contents.

This research comprehensively explored the different characteristics of social bots from humans and their impact on social media ecosystems and public opinion. Thus, it enables researchers and practitioners to understand the user persona of social bots in social media, to understand how they participate in the COVID-19 pandemic discussion, and to further consider how this artificial intelligent technology can be utilized to create a positive public health online environment.

## 7. Limitations

Firstly, we mainly focus on the data analysis of the characteristics and behavior patterns of social bots at the level of textual research. Research on the interaction mechanism between social bots and human behavior and the causal relationship behind it is not analyzed in-depth. Propaganda concepts, algorithm principles, power relations, and ethics also need to be further explored. Secondly, the amount of sample data is limited, making it difficult to present the social network of social bots in the COVID-19 pandemic discussion. We can continue to research the social network structure of social bots and model social bots’ role in the dissemination of public health information in the future.

## Figures and Tables

**Table 1:** COVID-19 pandemic topic tweet statistics.

Index	Social Bots	Humans	Total
**Tweets**	14,499	39,109	53,608
**Text length**	107	110	109
**Location**	Canada, United States, Australia, etc.	Canada, United States, United Kingdom, etc.	Canada, United States, United Kingdom, Australia, India, etc.

**Table 2:** Influence of social bot and human tweet accounts based on retweeted, liked, and replied.

	Social Bot Accounts (N = 10,098)			Humans Accounts (N = 35,492)		
	Account Number	Percent	Average Counts	Account Number	Percent	Average Counts
Retweetd (≠0)	9176	90.78%	1581.76	32,340	91.12%	1220.74
Liked (≠0)	8586	85.03%	2.14	3042	8.57%	3.05
Replied (≠0)	200	1.98%	0.02	720	2.03%	0.03
All (≠0)	60	0.59%	-	131	0.37%	-

## Data Availability

Not applicable.
